# Monoclonal Antibodies Targeting *Bacillus Calmette–Guérin* (BCG) Outer Membrane Proteins Confer Protection Against Tuberculosis

**DOI:** 10.1155/tbed/5410388

**Published:** 2026-10-16

**Authors:** Huoming Li, Yanyan Jiang, Chenxi Shi, You Zuo, Hao Li

**Affiliations:** ^1^ College of Animal Husbandry and Veterinary Medicine Jinzhou Medical University Jinzhou 121001 China jzmu.edu.cn; ^2^ Collaborative Innovation Center for Prevention and Control of Zoonoses Provincial Key Laboratory of Animal Product Quality and Safety Jinzhou Medical University Jinzhou 121001 China jzmu.edu.cn; ^3^ State Key Laboratory of Veterinary Public Health and Safety College of Veterinary Medicine China Agricultural University Beijing China cau.edu.cn

**Keywords:** antibody, BCG, *Mycobacterium bovis*, outer membrane proteins, tuberculosis

## Abstract

Tuberculosis (TB), caused by the *Mycobacterium tuberculosis* complex (MTBC), is a persistent global health crisis, remaining the world’s leading infectious killer. There were abundant outer membrane proteins (OMPs) distributed in the bacterial cell wall, while fewer proteins were identified and characterized as antigens that could induce protective antibodies against TB. The OMPs of BCG were extracted with the detergent N‐octyl‐β‐D‐glucopyranoside, and the extracted proteins were used to immunize mice to generate an antibody library. Subsequently, the library underwent HAT/HT screening, and five specific monoclonal antibodies (mAbs) targeting OMPs with higher titers named 9C5, 14E9, 9F6, 7B10, and 6F4 were obtained. Among the five mAbs, 9C5, 14E9, and 9F6 could increase macrophage phagocytosis, inhibit the mycobacteria growth in peripheral blood mononuclear cells (PBMCs), and enhance the fusion of phagosomes and lysosomes in macrophages. Mice were immunized with the three antibodies before being challenged with *M. bovis*, among which two antibodies, 14E9 and 9C5, exhibited a preventive effect against TB infection. Furthermore, antibody 9C5 also showed a therapeutic effect, characterized by significant decreases in lung burden and ameliorated lung lesions. The antigens targeted by the two antibodies 9C5 and 14E9 were identified by coimmunoprecipitation (co‐IP) combined with mass spectrometry (MS), and the results demonstrated that antibodies 9C5 and 14E9 recognize BCG_2760c and MPB83, respectively. This research promotes a better understanding of host protective humoral immunity by BCG vaccination and the roles of antibodies in TB vaccine design and development.

## 1. Introduction

The emergence of antibiotics has opened a new era in the anti‐TB field. Antibiotics were regarded as the most effective way of preventing and treating tuberculosis (TB), but new challenges have arisen, such as multidrug‐resistant TB (MDR‐TB) and extensively drug‐resistant TB (XDR‐TB) [1]. Therefore, more novel and effective prevention and treatment strategies against TB are urgently needed.

Antibodies from latent TB infection (LTB) patients can enhance phagolysosome maturation, inflammasome activation, and inhibit intracellular survival of Mtb [2]. The antibodies from highly exposed healthcare workers further demonstrated protection against TB in mice, and whole bacteria‐depleted antibodies abrogated the protective efficacy of these antibodies, which revealed that these antigens targeted by the above antibodies localized on the surface of Mtb [3]. Antibodies induced by surface antigens of bacteria mediated by professional phagocytes, such as macrophages and neutrophils, for eliminating invading pathogens presented antigens to lymphocytes, thereby activating the adaptive immune response [4]. Outer membrane proteins (OMPs), which are the antigens located on the outermost surface of bacteria, are widely recognized as promising vaccine candidates because of their surface exposure, high immunogenicity, and conservation across bacterial strains [5, 6]. A monoclonal antibody (mAb) named 06 mAb was generated from mice immunized with OMPs isolated from *Salmonella typhimurium* (*S. typhimurium*), which was proven to efficiently inhibit *S. typhimurium* infection [7]. A number of antigens are distributed on the surface of Mtb, including polysaccharides and proteins, and some components have been shown to induce protective antibodies against TB, such as LAM, MPB83, heparin‐binding hemagglutinin adhesin (HBHA), Acr, PstS1, and LpqH [8, 9]. Using bioinformatic analysis, 144 putative OMPs were listed based on the classical β‐barrel structure of the known crystal of MspA, a certain OMP in *Mycobacterium smegmatis* [10].

BCG is the only licensed human vaccine against TB and is widely used worldwide and can offer significant efficacy in preventing disseminated TB and TB meningitis in children [11]. BCG vaccination can induce long‐lived memory B‐cell and specific antibody responses against mycobacterial infections [12, 13]. Higher levels of antibodies against the Mtb antigen Ag85A correlate with a lower risk of TB disease in BCG‐vaccinated infants [14]. Moreover, encapsulated BCG vaccination can induce stronger and more protective immune responses characterized by higher levels of IgG2c antibodies [15]. Recent studies have shown that intravenous BCG conferred more effective immune protection in rhesus macaques compared to those immunized via subcutaneous or aerosol routes, and a significant association between IgM antibody titers in the blood and the reduction of bacterial load in the lungs of BCG‐immunized macaques was observed [16, 17]. These studies provide strong evidence that BCG vaccination can induce protective antibody responses against Mtb infections.

In this study, BCG OMPs were extracted and used to immunize mice for the establishment of an antibody library. After HAT/HT screening, the specific mAbs targeting OMPs were isolated and purified. Some antibodies with high affinity to BCG or *M. bovis* were selected and further characterized. The protective mAbs were determined in cell and mouse models. Furthermore, the antigens recognized by the protective mAbs were identified. This study provides insights into the protective antibody responses aroused by OMPs of BCG, which also provides new strategies for TB treatment and vaccine development.

## 2. Materials and Methods

### 2.1. Bacterial Strains and Cell Lines

In this study, two *Mycobacterium* species were used and cultured in 7H9 medium (BD) complemented with OADC (BD), including *Mycobacterium bovis* BCG *str. Pasteur* 1173P2 and *M. bovis* C68004 [18]. Cell lines J774a.1 were purchased from the National Infrastructure of Cell Line Resource (NICR), Beijing, China.

### 2.2. Bacterial and Cell Culture

Cell culture media, reagents, and antibiotics were purchased from Sigma–Aldrich, China. Macrophage serum free medium (MSFM) procured from Thermo Fisher Scientific, China, was used to culture peripheral blood mononuclear cells (PBMCs) from a mouse whole blood sample and for the mycobacterial growth inhibition assay (MGIA) in vitro. In this study, all bacterial cultures were grown and propagated at 37°C under a constant agitation of 90 rpm. BCG used to prepare OMPs was cultured in Sauton’s medium. All cell lines were cultured and propagated in the Dulbecco Modified Eagle’s medium (DMEM) supplemented with 10% fetal bovine serum (FBS) with appropriate antibiotics at 37°C with 5% CO_2_.

### 2.3. OMPs Preparation

A 500 mL culture of *M. bovis* BCG at mid‐log phase was inactivated by the addition of 100 μg/ml ciprofloxacin (Sigma) to the culture medium and incubated at 37°C for 1 h. Cells were harvested by centrifugation at 8000 rpm for 10 min at 4°C, followed by three washes with phosphate‐buffered saline (PBS). The bacterial pellet was resuspended in PBS supplemented with a 1 mM proteinase inhibitor cocktail. A 1/3 volume of glass beads was added to the bacterial solution, and cells were lysed by a BeadBug microtube homogenizer (Biospec) at 4000 rpm for 30 s. Bead beating was repeated 8 times with 1 min intervals on ice. Beads and cell debris were removed by centrifugation at 1000 rpm for 3 min.

The above cell lysate was sonicated at 300 W for 30 min with 2 s ON and 3 s OFF, followed by low‐speed centrifugation for 5 min at 2300 g to remove unbroken cells. Subsequently, the cell supernatant was subjected to centrifugation at 27,000 g for 1 h at 4°C, and this step was repeated once to obtain the final cell envelope. The cell pellet, corresponding to the cell envelope, was resuspended in PBS containing 1% (wt/vol) n‐octyl‐β‐D‐glucopyranoside (OBG; Sigma) and incubated for 1 h at 25°C. Then, samples were centrifuged twice at 27,000 g for 1 h at 4°C to separate the soluble from the insoluble fraction. The soluble fraction containing OMPs, supplemented with a proteinase inhibitor, was dialyzed overnight in PBS at 4°C to eliminate detergent OBG. The final soluble fraction was dissolved in 5× SDS loading buffer (250 mM Tris‐HCl [pH 6.8], 10% [wt/vol] SDS, 0.5% bromophenol blue, 50% [vol/vol] glycerol, 5% β‐mercaptoethanol) and boiled at 100°C for 10 min before SDS‐PAGE and Western‐blot (WB) analysis.

### 2.4. Mass Spectrometric Analysis

Extracted OMPs sample (56 μg) was mixed with 20 μL SDS loading buffer (1×) and boiled at 100°C for 10 min. The protein migration was performed with 10 μg of sample on a 1 mm thick of 12% SDS polyacrylamide gel (Invitrogen). After SDS‐PAGE analysis, the protein lanes were excised and divided into fractions according to a standard protein marker (Thermo Fisher). The gel pieces were washed with acetonitrile and digested with trypsin (Gibco) for 10–20 h at 37°C. The digested peptides were prepared for nano liquid chromatography‐tandem mass spectrometry (nano LC‐MS/MS) analysis as previously described [19], using the UniProt database for protein identification.

### 2.5. Generation of mAbs

Five female BALB/c mice (6–8 weeks old) were purchased from SI BEIFU Biotechnology Company, China. Animal experiments were approved by the Experimental Animal Welfare Ethical Review Consent of China Agricultural University (License Number AW71306202‐2‐01). The 25 µg OMP extraction from BCG was mixed with 50 µL of QuickAntibody‐Mouse 5 W adjuvant (Biodragon Immunotechnologies Ltd., Beijing, China) and immunized twice by intramuscular injection in the hind leg at 2‐week intervals. Two weeks after the final immunization, orbital blood samples were used to determine serum antibody titers by indirect ELISA. The booster immunization was administered to mice with the highest serum titers via the intraperitoneal (i.p.) route. Three days after the booster immunization, fusion of mice splenocytes with SP2/0 myeloma cells (3:1) was performed. Seeding fuzed cells into a 96‐well plate containing HAT medium and culturing for 7 days at 37°C with 5% CO_2_. Positive hybridoma cells were screened by ELISA following two rounds of subcloning by limiting dilution. All mAbs were generated by i.p. injection of positive cells into mice that had been pretreated with paraffin oil 1 week earlier. After 1 week, the mice with abdominal fluid were used to collect ascitic fluid, which were then purified using a protein G column (Genescript, L00209). Antibody purity and molecular weight were assessed by SDS‐PAGE analysis.

### 2.6. Indirect ELISA

The OMPs sample (0.1 µg/well) was coated onto a 96‐well plate and incubated overnight at 4°C to allow antigen absorption to the well walls. Each well was filled with 5% skim milk (200 µL/well) for 2 h at 37°C to reduce nonspecific binding. Subsequently, each well was added to hybridoma cell culture supernatant (100 µL/well) or purified mAbs, corresponding to the appropriate dilution, and incubated at 37°C for 1 h. After three washes with PBS containing 0.5% Tween 20 (PBST), horseradish peroxidase‐conjugated (HRP) goat anti‐mouse IgG (1:10,000) (Cwbio, 0102 S) was added to each well, and the plates were incubated at 37°C for 1 h. The serum from mice immunized with PBS served as the negative control. A 100 µL TMB chromogenic solution was used to develop the color at 37°C for 15 min, followed by stopping the reaction by adding an equal volume of 2 M sulfuric acid. The plates were placed on a multifunctional microplate reader (Tecan Spark) to detect the substrate reaction’s optical density (OD) at wavelength of 450 nm. Samples were deemed positive when the OD_450_ _nm_ of the lowest dilution was 2.1 times higher than that of the negative control.

### 2.7. Whole Bacterial ELISA

For testing binding between bacteria and antibody, mycobacterial species were cultured in 7H9 medium containing 10% OADC (BD) for 15 days and inactivated by heating at 80°C for 2 h. The bacteria were resuspended with distilled water and coated onto 96‐well plates (1 × 10^7^ CFU/well), followed by drying at 56°C. Subsequently, each well was added to 200 µL precooled methanol and incubated at 4°C for 2 h. After washing, the blocked manipulation was employed using 5% skim milk for 2 h at 37°C, and then the other process was deployed as described above.

### 2.8. Antibody Isotypes Determination

Antibody isotypes were determined using a mouse mAb isotyping kit (Frdbio, FRD90100K8) according to the manufacturer’s instructions. Briefly, the 96‐well plates were coated with OMP extraction (0.1 µg/well) of BCG and then incubated overnight at 4°C. The culture supernatant (100 µL/well) from hybridoma cells that secreted a specific antibody was added to each well, which was incubated for 1 h at 37°C. After three washes with PBST, HRP‐conjugated isotype secondary antibodies were diluted with PBST (1:500) and further incubated at 37°C for 1 h. Finally, TMB chromogenic solution was used to develop the color, and the antibody isotypes were determined based on the corresponding positive wells.

### 2.9. Phagocytosis by Flow Cytometry

FITC powder was dissolved and diluted in 0.1 M carbonate/bicarbonate buffer (pH 9.51). BCG grown in the log phase was collected by centrifuging at 4000 rpm for 10 min and stained with FITC solution at a final concentration of 1 mg/mL for 2 h. After three washes with PBS, a single‐cell bacterial suspension of BCG‐FITC was obtained by 25 passages through a 25‐gauge needle. BCG‐FITC (2 × 10^7^ CFU) was separately mixed with different mAbs (final concentration of 50 µg/mL) and incubated for 1 h at 37°C. The bacteria were centrifuged at 5000 rpm for 5 min to remove unbound antibody and resuspended in FBS‐free DMEM.

J774a.1 cells (2 × 10^5^ cells/well) with FBS‐free DMEM medium were cultured overnight in the 24‐well plates and then infected with BCG‐FITC (multiplicity of infection [MOI] of 10:1) for 3 h at 37°C with 5% CO_2_. Afterward, non‐internalized bacteria were removed by washing three times with prewarmed PBS. Cells were fixed with 4% paraformaldehyde (PFA) for 30 min at room temperature and resuspended in 500 µL 5% FCS/PBS. Then, all samples were analyzed by a BD Fortessa cytometer, following data processing using FlowJo software.

### 2.10. Intracellular Survival Assay

The J774a.1 cell line in log phase was resuspended in FBS‐free DMEM medium and plated into a 24‐well plate (2 × 10^5^ cells/well). Then, the cells were divided into the 0 h group and the 24 h group. PBS‐washed BCG (2 × 10^7^ CFU) was incubated with each antibody (final concentration 50 µg/mL) or PBS for 1 h at 37°C, followed by centrifugation at 8000 rpm for 3 min to remove the unbound antibody. These bacteria were used to infect coated cells with a MOI of 10 for 3 h, and extracellular bacteria were removed by washing with PBS three times. Meanwhile, the 24 h group cells were provided with 500 μL/well FBS‐free DMEM medium and then cultured for 24 h. In the 0 h group, the cells were lysed with 0.1% Triton X‐100 and diluted to be plated on the 7H10 agar supplemented with 10% OADC (BD). After 24 h, the 24 h group cells were lysed and plated as described above. All plates were cultured at 37°C for 3 weeks, and the bacterial counts were enumerated.

To determine the effect of FcγR, the cells (2 × 10^5^ cells/well) were blocked with 2 μg of anti‐CD16/CD32 antibody for 15 min prior to infection, followed by infection with BCG treated with serum or antibodies and incubated for 3 h at 37°C. All cells were lysed with 0.1% Triton X‐100 and diluted to be plated on the 7H10 agar supplemented with 10% OADC (BD), and then, phagocytosis was analyzed by GraphPad Prism software.

### 2.11. Confocal Microscopy

BCG was stained with 1 mg/mL FITC solution for 2 h, followed by three washes with PBS. The single bacterial suspension was obtained by 15 passages through a 25‐gauge needle. J774a.1 cells (1 × 10^5^ cells/well) were suspended in FBS‐free DMEM and seeded in 24‐well plates containing glass slides. Then, the cells were infected with prepared BCG‐FITC (with or without mAbs incubation) at a MOI of 10 for 3 h at 37°C. After three washes with prewarmed PBS, lysosome marker (Beyotime Biotechnology, C1046) was added to each well and incubated for 1 h at 37°C. The 4% PFA was used to fix cells at room temperature for 30 min. Slides were picked out and covered with 10 μL of anti‐fluorescence quenching reagent containing Hoechst 33342 reagents (Beyotime, P0133) and stored at 4°C. The confocal microscope (Nikon, A1HD25) was used to analyze phagosome‐lysosome fusion, and 10 distinct images were randomly captured at a magnification of ×100 (oil immersion lens). To exhibit the quantification of colocalization, a formula was used to evaluate the frequency of phagosome‐lysosome fusion, with the total number of cells and BCG phagosomes.

Colocalization of lysosome with BCG (%) $=\left[\sum_{i=1}^{n}\text{PS}÷\sum_{i=1}^{n}\text{TC}\right]\times 100\%$ (*n* = 10),

where PS refers to the total number of BCG phagosomes and TC refers to the total number of cells in 10 distinct images.

### 2.12. MGIA

We adapted the PBMCs‐based growth inhibition assay as previously described [18]. PBMCs were isolated using the Mouse Lymphocyte Isolation kit (Beyotime, C0029S) according to the manufacturer’s instructions. Briefly, we collected whole blood by cardiac puncture from C57BL/6J and then diluted it 1:1 with RPMI 1640 medium (Gibco), following which it was carefully layered over the surface of Lymphocyte Isolation Solution. The blood samples were centrifuged without brake at 500 g for 30 min at 20°C, and then the PBMCs layer was aspirated into a new centrifuge tube. Afterward, the PBMCs were washed three times with PBS at 250 g for 10 min and resuspended in MSFM (Gibco, 12065074).

PBMCs (2.5 × 10^5^ cells) were counted and plated into 24‐well plates, with a final concentration of 50 µg/mL mAbs or an equal volume of PBS. The PBS‐washed BCG or *M. bovis* was added to the cells at an MOI of 10 and incubated at 37°C for 4 days at 5% CO_2_, and then, the cells were lysed with 0.1% Triton X‐100 at 37°C for 10 min. Subsequently, cell lysates were diluted with PBST and plated onto 7H10 agar supplemented with 10% OADC (BD). All plates were cultured at 37°C for 3–4 weeks.

### 2.13. Passive Serum Transfer

To assess the immune protective effect of mAbs, all female C57BL/6J (6–8 weeks) were purchased and maintained under BSL‐3 conditions for 2 weeks. Afterward, the mice were divided into groups based on the treatment with mAbs or PBS. The experiment protocol was performed as previously described [18]. Briefly, each mouse was administered 500 µg mAbs or PBS via the i.p. route before *M. bovis* infection. After 5 h, the virulent *M. bovis* was used to infect each mouse with 200 CFU by the intranasal (i.n.) route and maintained for 2 weeks. Subsequently, all mice were euthanized, followed by an autopsy. The left lung was homogenized and plated on the 7H10 agar supplemented with 10% OADC (BD) and cultured at 37°C for 3 weeks until bacterial colonies developed. Additionally, the right lung and spleen were fixed with 10% formaldehyde solution for 1 week and then embedded in paraffin and sectioned into 3‐µm‐thick slices. All sections were subjected to hematoxylin and eosin (H&E) (Solarbio, G1120) staining after mounting on the glass and deparaffinization. All images were scanned by a Hamamatsu NanoZoomer‐S60 digital slide scanner and analyzed for various pathological lesion features.

Furthermore, the therapeutic effect of mAbs in vivo was also assessed using a mouse therapeutic model. Briefly, each mouse was first infected with 200 CFU of *M. bovis* by the i.n. route and after 24 h received therapy by the i.p. route with 500 µg mAbs. Then, the mAbs of the same dose were administered with a 1‐week interval 3 times. All mice were euthanized 1 week following the completion of final treatment, and the bacterial burden in the lungs was quantified, and the extent of pathological damage was evaluated as described above.

### 2.14. Coimmunoprecipitation Mass Spectrometry (Co‐IP/MS)

To verify the antigen, the technology of co‐IP was used to analyze the antibody against the surface antigen of BCG, with an immunoprecipitation kit (Beyotime, P2177). After magnetic beads were washed with TBS buffer, the beads were mixed with 50 µg/mL antibody and incubated at room temperature for 60 min. The bead‐conjugated antibody was washed with TBS buffer to remove unbound antibody, followed by incubation with the above BCG OMP at 4°C for 12 h. The lysis buffer containing the protease inhibitor reagent cocktail was used to elute the antibody‐beads‐antigen complex. All samples were analyzed with WB, and the identified antigen was determined by MS.

### 2.15. Statistical Analysis

Statistical analysis was performed using GraphPad Prism 9.5 software. Statistical significance was determined by one‐way ANOVA, followed by Tukey’s multiple comparisons test for comparisons among three or more groups. Results were interpreted as follows: ns: not significant;  ^∗^ *p*  < 0.05;  ^∗∗^ *p*  < 0.01;  ^∗∗∗^ *p*  < 0.001;  ^∗∗∗∗^ *p*  < 0.0001.

## 3. Results

### 3.1. OMP Preparation and Identification

The OMPs were separated from log‐phase BCG using differential centrifugation with n‐octyl‐β‐D‐glucopyranoside, and the protein concentration was measured to be ~0.7 mg/mL using the BCA reagent. SDS‐PAGE analysis of OMPs from BCG, which showed obvious bands ranging from 15 to 100 kDa (Figure 1A). Subsequently, the tryptic peptides were analyzed by LC‐MS/MS, and the resulting spectra were searched against the Mycobacterium bovis BCG proteome for protein identification. These proteins were classified with cell wall, cell membrane, cytoplasm, and unknown according to the subcellular location prediction from the UniProt database (https://www.uniprot.org/). Among them, 286 proteins were identified as cell membrane proteins, 231 proteins were identified as cytoplasm proteins, while only 49 proteins were localized on the cell wall. Importantly, the main body of the list location remained unknown (Figure 1B and Table S1). To verify the efficiency of extraction, the extraction samples were subjected to WB analysis, following analysis and characterization with antiserum against proteins of OMPs MctB and OMP A (OmpA), inner membrane protein FtsH, or cytosolic protein GroEL2, as shown in Figure S1. In spite of there being cytoplasmic composition contamination, which exhibited anticipated results containing certain OMP fractions of mycobacterial species, such as HBHA, MctB, and OmpA (Figure 1C–F). Next, the extracted samples were dialyzed using the PBS buffer to remove detergent component.

**Figure 1:**
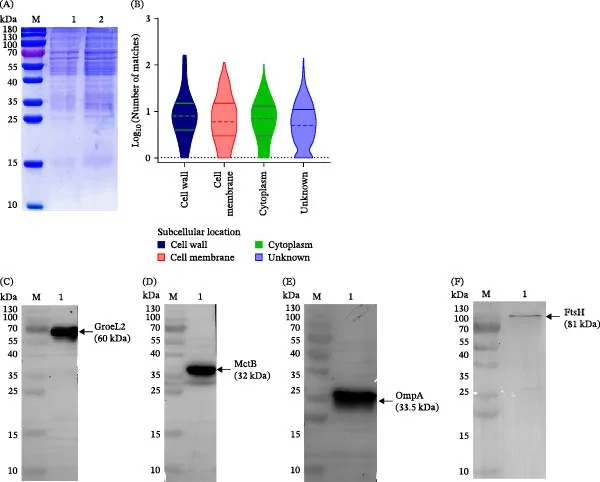
Preparation of OMPs sample. (A) OMPs analysis by SDS‐PAGE. M: protein prestained marker (0–180 kDa). Lane 1: 3.5 µg sample. Lane 2: 7 µg sample. (B) OMPs analysis by mass spectrometry. The gel contained OMPs samples that were excised and digested, following the mass spectrometry analysis. The MS data were shown as a violin plot: the vertical axis represented the peptide number of matches with the chromosome of BCG, while the horizontal axis represented the subcellular location of the protein. (C) Identified OMPs by GroEL2 serum. (D) Identified OMPs by MctB serum. (E) Identified OMPs by OmpA serum. (F) Identified OMPs by FtsH serum.

### 3.2. Generation of mAbs

The extracted OMPs mixed with the adjuvant were used to immunize with an optimal procedure. After immunization, the antibody titers of serum increased progressively with each booster dose of OMP antigens and finally reached 1:1,024,000 (Figure 2A). The CD16 and CD32 blocking could abrogate the increase of macrophage phagocytosis by the OMPs immunized serum (Figure 2B). The antibody isotypes in serum from immunized mice were analyzed by ELISA, showing a diverse isotype distribution, including IgG and IgM (Figure 2C). Subsequently, the hybridoma technology was employed to generate mAbs, and we successfully obtained 16 hybridoma cell lines that could stably secrete mAbs targeting OMPs (Table S2). Among them, the top five mAbs with the highest avidity for BCG and *M. bovi*s were selected for further characterization, namely, 9F6, 9C5, 14E9, 6F4, and 7B10, respectively (Figure 2F and Figure S2). The purified mAbs were analyzed using SDS‐PAGE, which showed clear heavy chains and light chains (Figure 2D). Meanwhile, the isotypes of antibodies were identified according to the manual instruction, of which 6F4, 9F6, and 14E9 were IgG2b, while 9C5 and 7B10 were IgG1 (Figure 2E). Additionally, these antibodies could bind to both the BCG and *M. bovis* strains, which suggested that the epitopes recognized by the mAbs were highly conserved between the two strains (Figure 2F).

**Figure 2:**
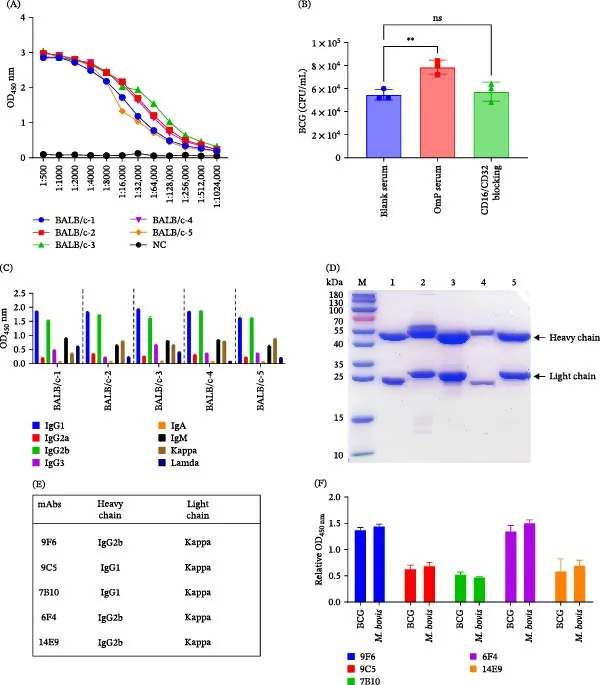
Preparation of antibodies against OMPs. (A) Serum titers of mice immunized with OMPs. A total of 25 µg OMPs were mixed with adjuvant, and then the complex was used to immunize mice via the hind leg muscle twice. The serum of mice was collected using orbital blood collection, and the titer was determined by ELISA. (B) OMPs serum‐mediated phagocytosis. J774A.1 cells (2 × 10^5^ cells/well) were applied to a 24‐well plate and maintained overnight, and serum was incubated together with BCG in cells for 3 h. Non‐internalized bacteria were removed with prewarmed PBS, and all cells were lysed with 0.1% Triton X‐100 and diluted to be plated on the 7H10 agar supplemented with 10% OADC (BD). All plates were cultured at 37°C for 3 weeks, and the bacterial counts were enumerated. The phagocytosis mediated by serum was analyzed using GraphPad Prism. (C) The analysis of antibody isotypes in serum. The plate coated with 0.1 µg OMPs sample was incubated overnight at 4°C, and the serum of mice immunized with OMPs was added to the well and incubated for 1 h. The secondary antibodies corresponding to the isotypes were added, the color was developed with TMB solution. The antibody isotype was determined based on the positive well. (D) Purification of antibodies. (E) Identification of purified antibodies’ isotypes. (F) Antibodies recognized whole bacteria. The plate was coated with hot‐inactivated whole bacteria (1 × 10^6^ CFU), BCG, or *M. bovis*, and blocked with 5% skim milk for 2 h. Purified antibodies diluted with PBST were added to each well and incubated at 37°C for 1 h. Then, the goat anti‐mouse IgG secondary antibody was added to each well. The TMB solution was utilized to develop color, and the sample well was designated as a positive well when the 450 nm value of the well was greater than that of the negative well.  ^∗∗^ indicates a statistically significant difference (*p* < 0.01) between the Blank serum and the Omp serum.

### 3.3. The Screening of Anti‐TB Protective mAbs in Vitro

The protective antibody‐mediated phagocytosis of macrophage cells has been well described in previous publications. Therefore, we developed a rapid screening protocol based on cytometry analysis, which could preliminarily identify antibodies that affected phagocytosis of macrophage cells. The BCG incubated with purified antibodies was used to infect J774a.1 cells, and these antibodies, such as 14E9, 9F6, and 9C5, were found to significantly enhance phagocytosis, while antibodies 6F4 and 7B10 either inhibited phagocytosis or had no effect (Figure 3A,B). Here, we first identified three antibodies that could facilitate the phagocytosis of macrophage cells.

**Figure 3:**
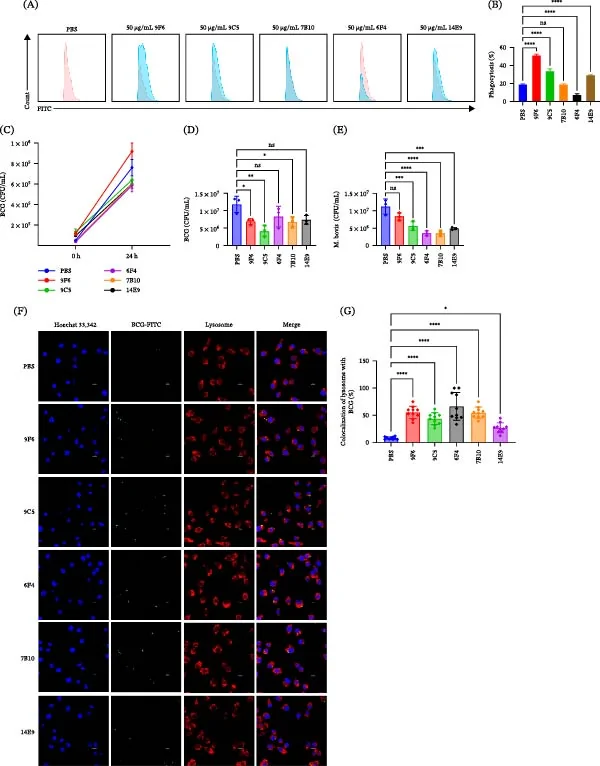
The protective antibody screening in vitro. (A) Phagocytosis analysis mediated by the individual antibody. The J774a.1 cells (2 × 10^5^ cells/well) were coated into 24‐well plates, and cultured overnight. The BCG‐FITC pretreated with mAbs or PBS was used to infect cells with a multiplicity of infections (MOI) of 10 for 3 h. These uninternalized bacteria were removed by prewarmed PBS washing three times, and 4% PFA was used to fix cells for 30 min at room temperature. The phagocytosis mediated by mAbs was analyzed through a flow cytometer. (B) The data statistics of subpart (A). All data were analyzed using FlowJo software. (C) Intracellular survival analysis in macrophage cells. The J774a.1 cells in log phase were cultured overnight in a 37°C incubator, and the BCG pretreated with antibodies or PBS was used to infect cells with an MOI of 10 for 3 h. After removing extracellular bacteria, the intracellular bacterial survival number was counted at 0 h and 24 h, respectively. (D) BCG growth inhibition in PBMCs. The PBMCs were isolated from the whole blood of mice, and coated into a 24‐well plate (2 × 10^5^ cells/well). The BCG was added into cells containing mAbs or an equal volume of PBS, and incubated in a 37°C incubator for 96 h. The surviving bacteria were counted after 2 weeks of culture at 37°C. (E) *M. bovis* growth inhibition in PBMCs. (F) The colocalization of BCG with lysosome. (G) The data statistics of subpart (F). The asterisks indicate levels of statistical significance as follows:  ^∗^ *p* < 0.05;  ^∗∗^ *p* < 0.01;  ^∗∗∗^ *p* < 0.001;  ^∗∗∗∗^ *p* < 0.0001 (compared with the PBS control group).

In addition, the number of BCG survivors in the J774a.1 cell was counted at 0 and 24 h, respectively. As a result, antibody‐mediated phagocytosis was again observed at 0 h for 9C5, 14E9, and 9F6. Importantly, these antibodies were also found to significantly restrict BCG duplication in cells (Figure 3C). Next, PBMCs were employed to further screen antibodies that could mediate mycobacterial intracellular growth. All antibodies could simultaneously inhibit both BCG and *M. bovis* intracellular growth except for 9F6 and 6F4, despite their displaying a variable inhibition effect (Figure 3D,E). Interestingly, the mAbs, 9F6 and 6F4, only exhibited inhibition in BCG or *M. bovis*, which might be attributed to the antigen’s exposure level.

One of the mechanisms by which antibody‐regulated intracellular growth inhibition occurs is the enhancement of phagocyte‐lysosome fusion. Therefore, confocal microscopy was used to assess lysosome‐bacteria fusion mediated by an antibody. The BCG was selected to be incubated with each antibody alone following infection with the J774a.1 macrophage. Compared to the PBS control, the antibodies, 9F6, 9C5, and 14E9, were found to significantly enhance phagosome‐lysosome fusion (Figure 3F,G). However, antibodies 7B10 and 6F4 did not show an apparent effect on phagocytosis, but they significantly enhanced phagosome‐lysosome fusion.

### 3.4. Protective Effect of mAbs in Vivo

The animal model was adopted to assess the preventive and therapeutic effects of anti‐TB. First, the above‐selected antibodies, 14E9, 9C5, and 9F6, which showed both enhanced phagocytosis and inhibited intracellular growth in vitro, were tested for preventive TB infection efficacy in vivo according to the optimal process (Figure 4A). The mice were challenged with *M. bovis* after being immunized with 500 µg of antibody for 5 h. No significant difference in organ coefficients of lungs and spleens was observed between mice treated with 14E9, 9C5, or 9F6 and those treated with PBS (Figure 4B,C). However, the bacterial load of lungs showed that preimmunized mice with antibodies 14E9 or 9C5 could decrease *M. bovis* colonization (Figure 4D). Then, pathological damage analysis of lungs from all groups was deployed according to Table S3, which did not present discrepancy compared to the PBS‐immunized group (Figure 4E and Table S4). Moreover, antibodies 7B10 and 6F4 were also tested for their preventive effects. No difference was observed in these data, neither lung burden nor pathological lesions (Figure S3). These results indicated that antibodies 14E9 and 9C5 possessed the capacity to prevent TB infection.

**Figure 4:**
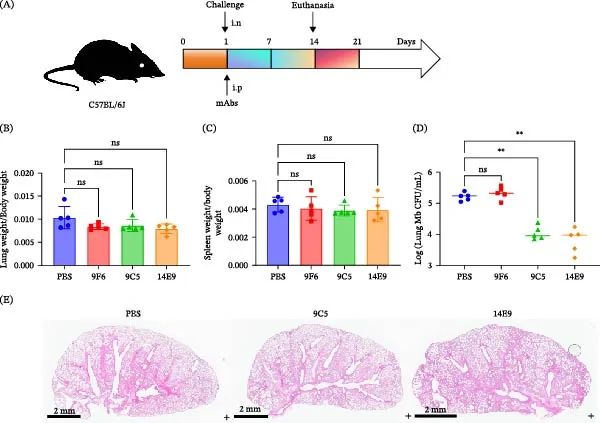
The antibody screening for preventing mice from TB infection. (A) The procedure of prophylactic effect identification of antibody. (B) The coefficient of lung of mice. (C) The coefficient of spleen of mice. (D) The bacterial burden of the lung of mice. (E) The pathological lesion analysis of mice.  ^∗∗^ indicates *p* < 0.01 compared with PBS group.

Furthermore, the mouse model infected with a low dose of *M. bovis* was used to examine the therapeutic capacity of antibodies, such as 14E9 and 9C5. The mice‐infected *M. bovis* were subjected to four times of antibody therapies via the i.p. route (Figure 5A). Behavioral changes and the weight of each group of mice were monitored during the treatment. Among them, the mouse body weight of the 9C5‐ and 14E9‐treated groups increased progressively with each booster therapeutic dose with 500 µg antibody compared to the PBS control group. Meanwhile, the organ coefficient of lungs and spleens from the group did not observe a significant decline compared to the PBS group (Figure 5B,C). Then, the lung burden and pathological lesions were also calculated to analyze the therapeutic effect, in which antibody, 9C5, was found not only to reduce lung burden but also to alleviate the lesions of the lung. Specifically, 9C5 could reduce lung load by 1 log compared with the PBS group, while 14E9 did not show a similar effect (Figure 5D). Subsequently, the lungs with pathological lesions, including organ injury and inflammatory cell infiltration, were evaluated using H&E staining based on the scoring matrix shown in Table S3. Then, the score values of each organ were recorded in Table 1. Mice treated with 9C5 showed milder pathological lesions, characterized by sparse inflammatory infiltrate, slight mucus hypersecretion, and more intact lung morphology, such as alveolar septa (+) (Figure 5E). However, more severe lung damage was observed in 14E9‐ and PBS‐treated mice, which was evidenced by obvious inflammatory infiltration, slight mucus hypersecretion, and alveolar septa thickening (++). Pathological analysis demonstrated that antibody 9C5 could ameliorate histopathological lesions in the lungs of mice caused by *M. bovis* infection. In total, the above results showed that mAbs 9C5 could decrease colonization and alleviate the organ lesions in the lung of mice infected intranasally with *M. bovis*.

**Figure 5:**
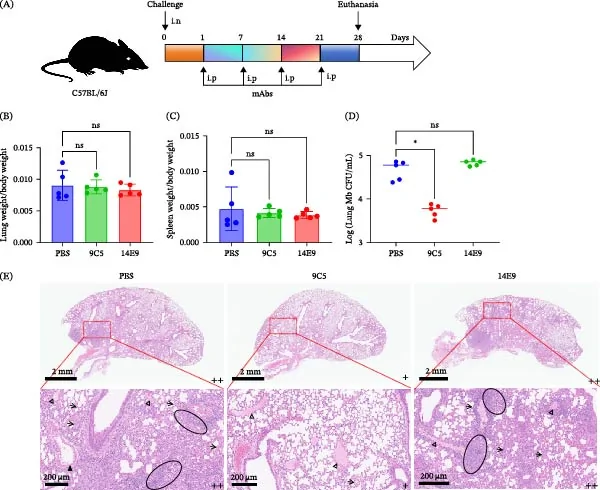
The therapeutic effect assessment of the antibody. (A) The procedure of therapeutic effect assessment in mice. (B) The coefficient of lung. (C) The coefficient of spleen. (D) The bacterial burden of the lung of mice. (E) The pathological lesion analysis of the lung.  ^∗^ indicates a statistically significant difference (*p* < 0.05) between experimental group and PBS group.

**Table 1:** The pathological lesions score from *M. bovis*‐infected mice of the therapeutic group.

Organs	Items	PBS	9C5	14E9
Lung	Inflammation	++	+	++
	Alveolar septal thickening	++	+	++
	Bronchial mucus exudation	+	+	+
	Stratification	++	+	++

### 3.5. Identification of Antigens

The antigen and epitope targeted by protective antibodies play an essential role in vaccine design. In the above results, we have demonstrated the protective ability of antibodies, 9C5 and 14E9, against TB infection. Here, some known surface antigen, such as MPB83, LpqH, PstS1, Acr, HBHA, MctB, and OmpA, were selected to exclude known protective antigens through ELISA. Interestingly, we found that antibody 14E9 could recognize the surface protein MPB83, for which the OD4_50_ value reached ~3‐fold that of the control group (Figure 6A). The specific binding was further identified by WB, showing the protein MPB83 as an antigen targeted by antibody 14E9 (Figure 6B). Additionally, the titer of antibody 14E9 to purified MPB83, BCG, and *M. bovis* reached 1:256,000, 1:16,000, and 1:32,000, respectively (Figure 6C).

**Figure 6:**
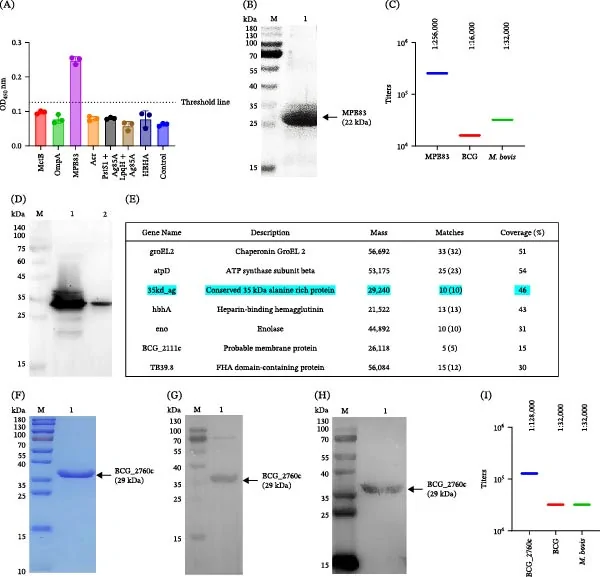
The identification of the antigen targeted by antibody 14E9. (A) The screening antigen recognized by 14E9 is a known surface protein. (B) The antibody 14E9 recognized MPB83. (C) The titers of antibody 14E9 to purified protein, BCG, and *M. bovis*. (D) The isolation of antigen targeted by 9C5. M: protein prestained marker (0–180 kDa). Lane 1: OMPs sample recognized by 9C5. Lane 2: target protein bound with 9C5. (E) The analysis of protein recognized by 9C5. (F) The expression of BCG_2760c. (G) The identification of BCG_2760c. (H) The 9C5 could specifically bind BCG_2760c. (I) The titers of antibody 9C5 to purified protein, BCG, and *M. bovis*.

At the same time, the antigen of antibody 9C5 was also identified through the above methods. Among these surface proteins, no obvious binding was observed for antibody 9C5 (Figure S4). The immunoblotting analysis indicated that antibody 9C5 could specifically bind to the crude OMP sample with a clear band, which was distributed between 25 and 35 kDa (Figure 6D). Next, the antigens recognized by antibody 9C5 were identified by co‐IP combined with MS analysis. The MS results of antibody 9C5 were listed, and the top seven proteins were expressed and verified with WB and ELISA (Figure 6E). Interestingly, GoreL2 appeared as the top protein in the MS data, while immunoblotting analysis excluded that it was a nonspecific binding (data not shown). Subsequently, systematic analysis was conducted on these remaining six proteins, following the confirmation that antibody 9C5 targeted BCG_2760c (Figure 6F–H).

The binding results of mAbs 9C5 against BCG, *M. bovis*, and the corresponding proteins (as a positive control) are presented in Figure 6I, in which the titers of antibody 9C5 reached 1:32,000, 1:32,000, and 1:128,000, respectively. Antibodies 9C5 and 14E9 exhibited excellent cross‐reactivity with BCG and *M. bovis* (Figure 2G). Furthermore, the cross‐reactivity of all antibodies against non‐TB bacillus strains was analyzed. The results demonstrated that these antibodies could reproducibly cross‐react with other bacterial strains, including *Mycobacterium kansasii* ATCC 12478 (*M_kansasii*), *Mycolicibacterium fortuitum subs*p. *fortuitum* DSM 46621 (*M_fortuitum*), *Mycobacterium colombiense* 10B (*M_colombiense*), and *Mycobacteroides abscessus* (*M_abscessus*), as shown in Figure S5. These findings suggested that antibodies, 14E9 and 9C5, possessed the ability to recognize shared epitopes on the surface of diverse *Mycobacterium* species and other non‐TB strains, indicating their potential as therapeutic agents for treating TB as well as some NTM infections.

## 4. Discussion

In this research, we selected OMPs derived from BCG as antigens and immunized mice to build a library of antibodies. Five specific antibodies targeted to OMPs were obtained and subjected to a screen for anti‐TB effects in vitro and in vivo. Among them, antibodies 9C5 and 14E9 were shown to not only inhibit mycobacterial intracellular growth but also reduce *M. bovis* colonization in the lungs of mice, and further data exhibited that antibody 9C5 possessed therapeutic potential for TB infection, presenting in decreased lung burden and ameliorating lung damage. The antigens targeted by the two antibodies were identified as MPB83 and BCG_2760c, respectively.

However, with respect to the abundance of antigens on the cell wall of mycobacterial species, there were still fewer antigens known to induce protective antibodies. Therefore, building an antibody library after immunity to OMPs has been considered as a choice to reveal more antigens [20]. The native hydrophobic characterization of membrane protein resulted in difficult extraction for OMPs of bacteria, while the emergence of detergent efficiently complements the defect. The nonionic detergent Triton 114 was used to extract the membrane protein of Mtb, which exhibited an excellent effect, with 1417 different proteins being identified [19]. Among them, 54 proteins were deemed as OMPs that contained a shared feature of the known OMP structure, such as signal peptide, amphiphilicity of the β‐strand region, and absence of hydrophobic α‐helices [10, 19]. Therefore, we used the above method to extract OMPs and then immunized mice. Regrettably, the serum titers of mice have not been found to show an obvious increase compared to the PBS‐treat group (data not shown). We speculated that the presence of detergent would inhibit the immune response of the host. Nevertheless, the detergent component, for example, Triton 114 and Triton X‐100, could not easily be removed because of the nature of lower critical micelle concentration (CMC). Therefore, a detergent, which not only could isolate the membrane protein but also remove it from extraction samples, has been an urgent need. As previously reported, N‐octyl‐β‐D‐glucopyranoside, a detergent possessing a higher CMC that could be removed by dialysis, could efficiently isolate OMPs of mycobacterial species. Here, N‐octyl‐β‐D‐glucopyranoside was selected as the extraction reagent to isolate the OMPs of BCG, followed by dialysis in PBS to remove the reagent [21]. The results indicated that these mice, which were immunized with OMPs following the aforementioned treatment, produced a significant increase in antibody titer.

The antigen targeted by antibody 9C5 was identified as BCG_2760c, while antibody 14E9 recognized MPB83. Interestingly, MPB83, a secreted mycobacterial lipoprotein, has been systematically documented as a protective antigen that has been applied in a subunit vaccine against TB [22, 23]. Importantly, previous research demonstrated that MPB83 could induce the host to produce the protective antibody, MBS43, against TB. Although the bacterial burden in mouse organs did not decrease, the antibody could alleviate the pathological lesion of the lung and prolong the survival rate of mice after *M. bovis* infection [22]. In our study, we found that the antibody 14E9, which targeted MPB83, also reduced the *M. bovis* colonization in the lung of mice. The divergent phenotypes between MBS43 and 14E9 were likely attributed to the difference in epitope specificity and experimental parameters. Epitope specificity was a critical determinant of antibody‐mediated protection. While both antibodies recognized MPB83, 14E9 reduced *M. bovis* lung colonization, whereas MBS43 did not affect bacterial burden, despite alleviating lung pathology [23]. This indicated that 14E9 may have targeted a functionally distinct epitope involved in bacterial survival or host–pathogen interaction, whereas MBS43 bound a nonneutralizing region. Indeed, the protective efficacy of anti‐lipoprotein antibodies was highly epitope‐dependent, as demonstrated for LpqH in *M. tuberculosis* [9]. Infectious route and model parameters further contributed to this discrepancy. Previous research employed an intravenous route, which bypassed mucosal immune barriers and altered antibody accessibility to pulmonary lesions. In contrast, aersol infection model better recapitulated natural transmission and granuloma microenvironments where secreted antigens like MPB83 were actively presented [24]. The route of infection profoundly influenced both bacterial dissemination kinetics and the local availability of target antigens for antibody neutralization [25, 26]. In the therapeutic infection model, mAb administration was performed via i.p. injection, in contrast to the i.n. route that was employed for *M. bovis* infection. The route was chosen for several reasons: (i) this route enables consistent and quantifiable delivery of mAbs, while i.n. administration is prone to uneven drug distribution and variable absorption efficiency as a result of mucociliary clearance [27]; (ii) i.p. injection allows antibodies to enter the systemic circulation through the peritoneal capillaries and lymphatic vessels, from where they can diffuse into lung tissues through the blood‐air barrier [28, 29]; (iii) previous studies have confirmed that systemically administered antibodies can effectively reach the lung parenchyma and provide protective effects against pulmonary pathogens [30, 31]. After entering systemic circulation, IgG antibodies can transudate into the alveolar space and opsonize *M. bovis*, thereby promoting Fc‐mediated phagocytosis and complement activation [32, 33]. This route also mimics the practical clinical scenario of passive immunotherapy, where intravenous or i.p. antibody infusion is more feasible than direct pulmonary delivery. In addition, we observed that the antibody 14E9 was capable of mediating phagocytosis of macrophages, enhancing lysosome fusion and inhibiting the BCG or *M. bovis* growth in PBMCs. These mechanisms have not been reported in antibodies induced by MPB83. There are various reasons that cause the different functions in these antibodies produced by MPB83. On the one hand, the abundance of the antigen might significantly affect the efficacy of the antibody. On the other hand, the extent of exposure to the antigen would directly decide the production of antibody. Additionally, the isotypes of antibody might also lead to different effects, which have been revealed in the concrete mechanism. For antibody targeting the same antigen epitope of HBHA, IgA isotypes exhibited excellent protective efficacy, while IgG isotypes did not [34].

Importantly, a protein encoded by gene BCG_2760c, which was highly 100% homologous with Rv2744c of *Mycobacterium tuberculosis* H37Rv (*M. tuberculosis* H37Rv) and *Mycobacterium bovis* AF2122/97 (*M. bovis* AF2122/97), was demonstrated to induce anti‐TB protective antibody. PspA, encoded by Rv2744c, an essential component of the Psp system, has been shown to regulate lipid droplet homeostasis and *M. tuberculosis* survival during nonreplicating persistence [35]. However, the whole bacterial ELISA analysis showed that the antibody 9C5 could admirably bind inactivated BCG or *M. bovis* (Figure 1). Here, PspA would traffic to the cell wall and cell membrane during modulating lipid homoeostasis, which could provide a window for antibody interaction [36]. As OmpA of *Acinetobacter baumannii*, it could induce mice to produce antibodies with potent bactericidal effects, along with a decrease in bacterial adherence, internalization, and proliferation in epithelial cell lines [37, 38]. PspA also exhibited high to both mycobacterial species and nontuberculous mycobacteria, and antibody 9C5 could bind these bacterial strains, which provided a reference for broad‐spectrum vaccine design. Other bacterial OmpA, including *E. coli* and *M. bovis*, also displayed inhibited bacterial infection capacity [18, 39]. Moreover, PspA has been proposed as a protective antigen in *Streptococcus pneumoniae* (*S. pneumoniae*), and fusion of PspA to pneumolysin showed the enhancement of vaccine efficacy through inducing complement C3 deposition on the surface of multiple pneumococcal strains [40–42]. Furthermore, the reverse genetic technology was employed to replace the neuraminidase of influenza A with PspA, which constructed a bivalent vaccine that could protect mice from lethal challenge infection of influenza A and *S.pneumoniae* [43].

In this study, despite revealing some protective antigens, these quantities also remain lower compared to the ample protein magnitude distributed in the mycobacterial chromosome. This might be caused by multiple reasons, including the library capacity of the antibody, antigen quality, and screen protocol. Despite identifying these protective antibodies, these antibodies have only a limited application scope because they are all murine antibodies. The category of humanized modification of all antibodies was deployed to expand their application. In addition, the underlying mechanism of these protective antibodies remains unclear in vitro and in vivo. Therefore, it would be worthwhile in future research to focus on these antibodies, whether to specifically target some region or mediate other immune cells to control TB infection.

## Author Contributions

Hao Li conceived the study and directed the research. Hao Li and Huoming Li acquired funding, designed the experiment, and wrote the manuscript. Huoming Li performed the experiments and analyzed the data. Yanyan Jiang, Chenxi Shi, and You Zuo assisted with the experiments.

## Funding

This work was supported by the National Natural Science Foundation of China (Project Number 32070937), the National Major Science and Technology Projects (Project Number 2026ZD01913003), the Department of Science and Technology of Liaoning Province (Project Numbers 2025‐BLSH‐127 and 2023‐MSLH‐037), and the Young Scientific and Technological Talent Support Program of Jinzhou Medical University (Project Number JYQT202504).

## Ethics Statement

All animal experiments were conducted in accordance with institutional guidelines and approved by the Institutional Animal Care and Use Committee (Approval Number AW71306202‐2‐01).

## Consent

The authors have nothing to report.

## Conflicts of Interest

The authors declare no conflicts of interest.

## Supporting Information

Additional supporting information can be found online in the Supporting Information section.

## Supporting information


**Supporting Information** The supporting file contains Tables S1–S4 and Figures S1–S5, providing the results of the mass spectrometry analysis of the BCG OMPs (Table S1), the preparation and affinity of the OMP‐specific antibodies (Figures S1–S2; Table S2), the assessment of their prophylactic effect in mice (Figure S3; Table S4), the identification of the antigen targeted by the protective antibody 14E9 (Figure S4), their cross‐reaction with nontuberculous mycobacteria (Figure S5), and the scoring criteria for lung pathological lesions (Table S3). The pathological lesion scores of *M. bovis*‐infected mice in the prophylactic group (Table S4).

## Data Availability

All data generated or analyzed during this study are included in this published article (and its supporting information files).
