# Supplementary material for: Monoclonal Antibodies Targeting Bacillus Calmette–Guérin (BCG) Outer Membrane Proteins Confer Protection Against Tuberculosis
Source: Transbound Emerg Dis. 2026 Oct 16;2026:5410388. doi: 10.1155/tbed/5410388 (PMC13673303; doi:10.1155/tbed/5410388)
Supplement: Supplementary file 1 [file TBED-2026-5410388-s001.docx]

Figure S1. Preparation of identified serum against OMPs. A. Amplification of gene fraction. M: Marker (5000 DL marker) , Lane 1: GroeL2, Lane 2: MctB, Lane 3: OmpA, Lane 4: FtsH. B. Purification of proteins. M: Marker (0-180 kDa) , Lane 1: GroeL2, Lane 2: MctB, Lane 3: OmpA, Lane 4: FtsH. C. Identification of protein by HIS antibody. M: Marker (0-180 kDa) , Lane 1: GroeL2, Lane 2: MctB, Lane 3: OmpA, Lane 4: FtsH. D. Identified GroeL2 protein by serum. E. Identified MctB protein by serum. F. Identified OmpA protein by serum. G. Identified FtsH protein by serum.

Table S2. Specific monoclonal antibodies from mouse immunized with OMPs of BCG.

| **mAbs** | **Heavy chain** | **Light chain** |
| --- | --- | --- |
| 1B4 | IgG2a | Kappa |
| 1E7 | IgG1 | Kappa |
| 7B10 | IgG1 | Kappa |
| 5A11 | IgG1 | Kappa |
| 14E9 | IgG2b | Kappa |
| 5F10 | IgG1 | Kappa |
| 9C5 | IgG1 | Kappa |
| 6F4 | IgG2b | Kappa |
| 1G7 | IgG2a | Kappa |
| 4B3 | IgG2a | Kappa |
| 5A9 | IgG2b | Kappa |
| 6E3 | IgG2b | Kappa |
| 6H10 | IgG2a | Kappa |
| 4H3 | IgG1 | Kappa |
| 9F6 | IgG2b | Kappa |
| 5F11 | IgG1 | Kappa |

Figure S2. The affinity of OMPs specific antibodies with BCG or *M.bovis*.

Table S3. The scoring criteria for evaluating the pathological lesion in lung of mice.

| **Lung** | | | |
| --- | --- | --- | --- |
| **Inflammation** | | **Alveolar septal thickening** | |
| 0 | None | 0 | None |
| 1 | Sparse inflammatory infiltrate | 1 | Slight thickening |
| 2 | Mild inflammatory infiltrate | 2 | Mild thickening |
| 3 | Multiple inflammatory lesions | 3 | Moderate thickening |
| 4 | Severe inflammatory infiltrate | 4 | Severe thickening |
| **Bronchial mucus exudation** | |  |  |
| 0 | None |  |  |
| 1 | Slight mucus exudation |  |  |
| 2 | Mild mucus exudation |  |  |
| 3 | Moderate mucus exudation |  |  |
| 4 | Severe mucus exudation |  |  |

Figure S3. The prophylactic affect assessment of antibody. A. The co-efficient of lung. B. The coefficient of spleen. C. The bacterial burden of the lung.

Table S4. The pathological lesions score from *M.bovis* infected mice of prophylactic group.

| Organs | Items | PBS | 9C5 | 14E9 |
| --- | --- | --- | --- | --- |
| Lung | Inflammation | + | + | + |
|  | Alveolar septal thickening | + | + | + |
|  | Bronchial mucus exudation | + | + | + |
|  | Stratification | + | + | + |

Figure S4. The identification of antigen targeted by protective antibody. A. The known surface protein in mycobacterial species’ cell wall. M: protein prestained marker (0-180 kDa). Lane 1: HBHA. Lane 2: LpqH-Ag85A. Lane3: PstS1-Ag85A. Lane 4: Acr. Lane 5: OmpA. Lane 6: MctB with deleted N-terminal 90 base. Lane 7: MPB83. B. The antigen screening of antibody 9C5.

Figure S5. The cross-reaction of the antibody in non-tuberculosis.
